# Immune checkpoint blockade: releasing the breaks or a protective barrier to COVID-19 severe acute respiratory syndrome?

**DOI:** 10.1038/s41416-020-0930-7

**Published:** 2020-06-16

**Authors:** Oliver J. Pickles, Lennard Y. W. Lee, Thomas Starkey, Luke Freeman-Mills, Anna Olsson-Brown, Vinton Cheng, Daniel J. Hughes, Alvin Lee, Karin Purshouse, Gary Middleton

**Affiliations:** 1 Institute of Immunology and Immunotherapy, University of Birmingham, Edgbaston, Birmingham B15 2TT UK; 2 Institute of Cancer and Genomic Sciences, University of Birmingham, Edgbaston, Birmingham B15 2TT UK; 3 Wellcome Centre for Human Genetics, University of Oxford, Oxford, OX3 7BN UK; 4 Clatterbridge Cancer Centre, Bebington, Wirral CH63 4JY UK; 5 Institute of Translational Medicine, University of Liverpool, Liverpool, L69 3GL UK; 6 Leeds Cancer Centre, St James’s University Hospital, Leeds, LS9 7TF UK; 7 School of Biomedical Engineering and Imaging Sciences, King’s College London, London, SE1 7EH UK; 8 UCL Cancer Institute, University College London, London, WC1E 6BT UK; 9 Edinburgh Cancer Research Centre, Western General Hospital, Edinburgh, EH4 2XR UK

**Keywords:** Cancer immunotherapy, Cancer

## Abstract

The rapid emergence of COVID-19 has sent shockwaves through healthcare systems globally, with cancer patients at increased risk. The interplay of the virus and host immune system has been implicated in the development of ARDS. Immunotherapy agents have the potential to adversely potentiate this phenomenon, requiring careful real-world observation.

## Main

The last decade has seen the emergence of immunotherapy within oncology. Immune checkpoint blockade (ICB), targeting cytotoxic T-lymphocyte-associated protein 4 (CTLA-4) and the programmed cell death 1 (PD-1)/programmed death ligand 1 (PD-L1) axis, is an established standard of care in treating many tumours. Increasingly, combination regimes involving both PD-1 and CTLA-4, or combination with other treatment modalities including radiotherapy and chemotherapy are being utilised.^1^ However, immunotherapies have a distinct toxicity, with predominantly autoimmune side effects. The spread of coronavirus disease 2019 (COVID-19) has sparked unprecedented international concern.^2^ But the associated risk of COVID-19 for patients on ICB is unclear. This comment summarises our preliminary knowledge of the interaction between ICB and COVID-19. We discuss the molecular biology of acute respiratory distress syndrome (ARDS), and its relationship to the severe acute respiratory syndrome coronavirus 2 (SARS-CoV-2). We speculate on the impact of ICB therapy on this pathological process. We identify further issues with ICB in the context of a COVID-19 pandemic. Finally, we recommend a unified response to the crisis by British oncologists, under the aegis of the UK Coronavirus Cancer Monitoring Project (UKCCMP).

### Immunology of ARDS and coronavirus response

The deterioration of many patients with COVID-19 is driven by an immune-mediated cytokine release syndrome (CRS) and the associated ARDS.^3^ This is a form of respiratory failure characterised by widespread rapid onset inflammation in the lungs. The immunology of ARDS is complex. Many of the inflammatory cytokines and chemokines linked to host viral defence have also been implicated in the pathogenesis of the syndrome. Higher plasma and alveolar concentrations of tumour necrosis factor (TNF)-α, interleukin (IL)-1β, IL-6, and IL-8 have all been linked to poorer outcomes.^4^ Tissue-resident macrophages are the likely source of the immune response, with resultant chemokine secretion leading to tissue ingress of peripheral immune cells including neutrophils and lymphocytes.^5,6^

The emerging serological data on SARS-CoV-2 patients resemble this classical ARDS picture. Higher levels of IL-2, IL-7, IL-10, granulocyte colony- stimulating factor, interferon γ-induced protein 10, monocyte chemoattractant protein-1, macrophage inflammatory protein, and TNF-α were all seen in patients requiring ITU admission.^7^ Furthermore, analysis of T-cell populations in a case of fatal COVID-19 ARDS revealed an activated CD8+ phenotype, with increased granulysin and perforin positivity.^8^ Elevated IL-6 plasma levels have been linked to worse prognosis overall and early phase clinical trials have been launched for the anti-IL-6 drugs tocilizumab (ChiCTR2000029765), siltuximab (NCT04322188) and sarilumab (NCT04324073).^9,10^

### ARDS and immune checkpoint blockade drugs

Immune checkpoints have evolved primarily as a mechanism of preventing injury to healthy tissue from overzealous immune attack, providing a delicate balance with effective pathogen control versus organ collateral damage. Up-regulation of both the PD-1 axis and CTLA-4 is seen in response to acute and chronic infections, and the manipulation of checkpoint signalling has shown early promise in improving responses to enduring infections including malaria and HIV.^11^

Whilst an alteration in viral susceptibility with ICB is theoretically possible, our concern is that ICB may potentiate ARDS. Indeed, the cytokine profile of ARDS would normally be seen as a desirable immune response against tumours.^12^ Although rare, cases of CRS have been reported following anti-PD-1 monotherapy, though are more typical with chimaeric antigen receptor (CAR) T-cell therapies. These cases are notably felt to be IL-6 driven, responding to the anti-IL-6 drug tocilizumab.^13^ IL-6 plasma levels are unfavourable prognosticators in COVID-19, therefore there is potential that ICB may lead to more severe immune hyperactivation or increased incidence of ARDS in COVID-19 patients.

### Additional problems

Beyond the worrying link between ARDS and ICB biology, ICB delivery during the COVID-19 pandemic has three additional problems.

First, the risk of merely attending a hospital environment for therapy delivery. The nature of oncology outpatient clinics has had to change dramatically to minimise the transmission risks of busy waiting rooms and multiple healthcare worker interactions.^14^

Second is the diagnostic dilemma posed by ICB toxicity. Significant pneumonitis is a relatively uncommon ICB side effect (Grade 3/4 in 1–2%), although is an important cause of ICB treatment-related mortality. However, the radiological features of ICB-related pneumonitis are varied and non-pathognomonic, with important differentials including infection and, in the current climate, SARS-CoV-2 infection.^15^ ICB-related pneumonitis might therefore be mistaken for COVID-19.

Third are the implications for the management of such pneumonitides, or other immune-related adverse events (irAEs). The mainstay of therapy is high-dose steroids followed by further immunosuppressive agents, including anti-TNFs.^15^ The use of steroids has been controversial so far in the treatment of SARS-CoV-2, with conflicting evidence. At present, the World Health Organization (WHO) advises against the administration of steroids to patients with suspected COVID-19, although there may be a role in critically unwell patients with ARDS, however, the evidence base for this is beyond the scope of this comment. The WHO has also recommended against the discontinuation of non-steroidal disease-modifying agents in gastroenterological, rheumatological and dermatological conditions. In conditions where steroids are clearly indicated they should continue to be administered.^16^

### Existing Chinese epidemiology

We have a paucity of data to guide the use of ICB therapy during the COVID-19 pandemic. Two Chinese reports are welcome but limited in scope. Liang et al.^17^ suggested a higher overall incidence of COVID-19 in cancer patients (1% versus 0.29% general population), as well as an increased severity of infection. However, only 18 cancer patients were included in this study and most were cancer survivors in long-term follow-up, with no recent systemic anti-cancer therapy, with only one patient receiving an unspecified immunotherapy.^17^ Zhang et al.^18^ retrospectively analysed a further cohort of 28 SARS-CoV-2-infected cancer patients from Wuhan, China. An increased risk of severe infection was once again noted in patients receiving systemic therapy in the 14 days prior to presentation; however, only one patient had received immunotherapy in combination with chemotherapy.^18^

The overall number of patients in these studies is small, and resultant analysis is likely to remain speculative at this stage. Moreover, we cannot turn to previous coronavirus outbreaks to guide us. In 2003, SARS-CoV spread and was contained prior to the development of ICB. Likewise, very few patients received ICB treatment during the 2012 Middle East respiratory syndrome coronavirus (MERS-CoV) outbreak. Additionally, the scale of the outbreak and global impact from both SARS-CoV and MERS-CoV was less severe.^19^

### The International Oncology Reaction and the UKCCMP

In the UK, the National Institute for Health and Care Excellence (NICE) has produced a guideline for the systemic treatment of cancer patients during the COVID-19 pandemic.^14^ Most of this guideline focuses on pragmatic management strategies that many readers will already recognise as happening within their centres, including minimising risk to patients from hospital encounters. Treatments should be assigned a priority level, reflecting the overall benefit likely from treatment. Immunotherapy treatments are generally likely to be in favourable priority groups. NICE has therefore recommended, rather than a total halt to immunotherapy, adapted rotas, including reducing the frequency of dosing from every 4 weeks to every 6 weeks. This will help to minimise hospital visits and resource use.^14^

Many oncologists are taking a cautious approach, and, where possible, may look to suspend immunosuppressive treatment where this is felt safe to do so. This may be secondary to the reasonable anxieties outlined above, and to the dearth of specific data. We suggest that greater national co-ordination is required. With concerted evidence gathering, more specific clinical guidance could be generated. To this end, the UKCCMP (see https://ukcoronaviruscancermonitoring.com), launched on 18 March 2020 and is aiming to involve over 90% of UK cancer centres. A Local Emergency Response Reporting Group has been created at each UK cancer centre to ensure continued updating of the UKCCMP. The project will collect data on SARS-CoV-2-positive cancer patients, including present cancer treatment and clinical outcomes, enabling oncologists to gain crucial insights to inform decision making with regards to immunotherapy.^20^

The COVID-19 pandemic continues to evolve and to apply unprecedented pressures to healthcare systems globally. There is limited experience of how ICB will alter the clinical course of COVID-19 infection, although there are clear mechanistic and biological features related to the development of CRS and ARDS that could be deleterious to our patients. An urgent contemporaneous collection of real-world oncology COVID-19 patient outcomes is urged to allow evidence-based recommendations.

## Data Availability

Not applicable.

## References

[1] Kruger, S., Ilmer, M., Kobold, S., Cadilha, B. L., Endres, S., Ormanns, S., et al. Advances in cancer immunotherapy 2019 – latest trends. *J. Exp. Clin. Cancer Res*. **38**, 10.1186/s13046-019-1266-0 (2019). 10.1186/s13046-019-1266-0 PMC6585101 31217020

[2] Lu R, Zhao X, Li J, Niu P, Yang B, Wu H (2020). Genomic characterisation and epidemiology of 2019 novel coronavirus: implications for virus origins and receptor binding. Lancet.

[3] Mehta P, McAuley DF, Brown M, Sanchez E, Tattersall RS, Manson JJ (2020). COVID-19: consider cytokine storm syndromes and immunosuppression. Lancet.

[4] Han S, Mallampalli RK (2015). The acute respiratory distress syndrome: from mechanism to translation. J. Immunol..

[5] Tseng CT, Perrone LA, Zhu H, Makino S, Peters CJ (2005). Severe acute respiratory syndrome and the innate immune responses: modulation of effector cell function without productive infection. J. Immunol..

[6] Meduri GU (1997). Host defense response and outcome in ARDS. Chest.

[7] Huang C, Wang Y, Li X, Ren L, Zhao J, Hu Y (2020). Clinical features of patients infected with 2019 novel coronavirus in Wuhan, China. Lancet.

[8] Xu Z, Shi L, Wang Y, Zhang J, Huang L, Zhang C (2020). Pathological findings of COVID-19 associated with acute respiratory distress syndrome. Lancet Respiratory Med..

[9] Ruan Q, Yang K, Wang W, Jiang L, Song J (2020). Clinical predictors of mortality due to COVID-19 based on an analysis of data of 150 patients from Wuhan, China. Intensive Care Med..

[10] Zhang, C., Wu, Z., Li, J.-W., Zhao, H., Wang, G.-Q. The cytokine release syndrome (CRS) of severe COVID-19 and Interleukin-6 receptor (IL-6R) antagonist Tocilizumab may be the key to reduce the mortality. *Int. J. Antimicrob. Agents.*10.1016/j.ijantimicag.2020.105954 (2020). 10.1016/j.ijantimicag.2020.105954 PMC7118634 32234467

[11] Wykes MN, Lewin SR (2018). Immune checkpoint blockade in infectious diseases. Nat. Rev. Immunol..

[12] Qu, X., Tang, Y., Hua, S. Immunological approaches towards cancer and inflammation: a cross talk. *Front. Immunol.* **9**, 10.3389/fimmu.2018.00563 (2018). 10.3389/fimmu.2018.00563 PMC5890100 29662489

[13] Rotz, S. J., Leino, D., Szabo, S., Mangino, J. L., Turpin, B. K., Pressey, J. G. Severe cytokine release syndrome in a patient receiving PD-1-directed therapy. *Pediatr. Blood Cancer.* **64**, 10.1002/pbc.26642 (2017). 10.1002/pbc.26642 28544595

[14] National Institute for Health and Care Excellence. COVID-19 rapid guideline: delivery of systemic anticancer treatments. NICE guideline [NG161]. https://www.nice.org.uk/guidance/ng161 (2020). 33497155

[15] Haanen J, Carbonnel F, Robert C, Kerr KM, Peters S, Larkin J (2017). Management of toxicities from immunotherapy: ESMO Clinical Practice Guidelines for diagnosis, treatment and follow-up. Ann. Oncol..

[16] World Health Organization. Clinical management of severe acute respiratory infection when COVID-19 is suspected. https://www.who.int/publications-detail/clinical-management-of-severe-acute-respiratory-infection-when-novel-coronavirus-(ncov)-infection-is-suspected (2020).

[17] Liang W, Guan W, Chen R, Wang W, Li J, Xu K (2020). Cancer patients in SARS-CoV-2 infection: a nationwide analysis in China. Lancet Oncol..

[18] Zhang L, Zhu F, Xie L, Wang C, Wang J, Chen R (2020). Clinical characteristics of COVID-19-infected cancer patients: a retrospective case study in three hospitals within Wuhan, China. Ann. Oncol..

[19] de Wit E, van Doremalen N, Falzarano D, Munster VJ (2016). SARS and MERS: recent insights into emerging coronaviruses. Nat. Rev. Microbiol.

[20] The UK Coronavirus Cancer Monitoring Project team. The UK Coronavirus Cancer Monitoring Project: protecting patients with cancer in the era of COVID-19. *Lancet. Oncol.*10.1016/s1470-2045(20)30230-8 (2020). 10.1016/S1470-2045(20)30230-8 PMC7159870 32304634

